# Ultrasound versus manipulation to determine an intercostal space for single-port thoracoscopy surgery: a diagnostic accuracy study

**DOI:** 10.1186/s12957-020-01870-3

**Published:** 2020-05-23

**Authors:** Chenxi Li, Jianjun Wang, Zeheng Ma, Bing Li, Kang Kang, Li Wei, Wei Zhang

**Affiliations:** 1 Department of Anesthesiology and Perioperative Medicine, Henan Provincial People’s Hospital, No. 7, Weiwu Road, Zhengzhou City, 450003 Henan Province China; 2 Department of Thoracic Surgery, Henan Provincial People’s Hospital, No. 7, Weiwu Road, Zhengzhou City, Henan Province China

**Keywords:** Intercostal space, Positioning, Ultrasound, Manipulation

## Abstract

**Background:**

Malposition of the intercostal space used for single-port thoracoscopy surgery can lead to problems. This study was to assess the accuracy of point-of-care ultrasound in verifying the position of intercostal space.

**Methods:**

A total of 200 patients, ASA (American Society of Anesthesiologists) physical status I or II, who underwent single-port thoracoscopic lobectomy, were enrolled. After the induction of anesthesia, a thoracic team confirmed the incision position. Firstly, the intercostal space was located by a young resident thoracic surgeon by ultrasound. Secondly, the intercostal space was located by an experienced thoracic surgeon by manipulation. Finally, the investigator verified the location of the intercostal space under direct vision through thoracoscopy, which was recognized as standard method. The time required by ultrasound and manipulation were recorded.

**Results:**

The inter-relationships between ultrasound and the standard method and between manipulation and the standard method were consistent. Manipulation positioning showed a sensitivity of 90.6% and specificity of 30% while ultrasound positioning showed a sensitivity of 87.1% and specificity of 60%. The specificity of ultrasound positioning was higher than that of manipulation position. The time required by ultrasound was shorter than that required by manipulation.

**Conclusions:**

Compared with the manipulation method, the ultrasound-guided method could accurately locate the intercostal space. Ultrasound requires less time than manipulation.

**Trial registration:**

ISRCTN10722758. Registered 04 June 2019

## Background

Single-incision or single-port procedures, such as lobectomy, characterized by less pain and shorter length of hospital stay, have gained increasing attention with the development of endoscopic devices and techniques [1, 2]. Since all the instruments are placed in a single small port, the accuracy of locating the intercostal space plays a very important role in surgery, and proper positioning of this port is essential [3]. The corresponding incision location was selected for different lesion sites [4]. Malposition of the intercostal space could lead to the following problems: increased difficulty of the surgical operation, prolonged operation time, and even the requirement of another new port.

The accuracy of traditional manipulation positioning relies on the rich clinical experience of the surgeon. Considering the anatomical variations, gender, and body mass indexes of patients, manipulation positioning tends to be subjective and uncertain. With the recent popularization of point-of-care ultrasound, an increasing number of clinical practices have been increasingly dependent on ultrasound [5, 6]. The accuracy of locating the intercostal space relies on the effective positioning of the ribs. The ribs need to be clearly felt by the hand to accurately locate the intercostal space in traditional manipulation positioning. In some cases, however, the ribs could not be identified clearly [7–9]. In contrast, the ribs can be clearly and easily shown with ultrasound: the cortex of the rib is strongly echogenic, and the posterior periosteum is hypoechoic [10]. The ribs can be displayed in real time under ultrasound, thus avoiding subjectivity and empiricism [11].

Whether ultrasound can be used to locate intercostal spaces is unclear. The purpose of this study was to evaluate and compare the accuracy of ultrasonography and manipulation to locate an intercostal space for single-port thoracoscopic surgery.

## Methods

### Participants

This study was approved by the Medical Ethics Committee of Henan Provincial People’s Hospital. Written informed consent and information release approvals were obtained from all patients prior to their participation in the study. The clinical trial registration code was ISRCTN10722758. The study protocol complied with the 1975 Declaration of Helsinki.

This study was performed at the Henan Provincial People’s Hospital. After gaining approval, 200 subjects were enrolled in this study from June 2019 to September 2019. The inclusion criteria are as follows: aged 18 to 65 years, elective single-port thoracoscopic lobectomy, and willingness to participate. The exclusion criteria are as follows: a history of chest wounds or infections, subcutaneous emphysema, and changed surgical schedule.

### Study protocol

#### Overview

To guarantee the blinding principle of this study, 4 researchers were included in the study. One was the primary anesthesiologist (PR), who was one of the attending faculty members of the department, was responsible for patient care throughout the study and was able to view all of the patients’ vital signs and terminate the study protocol if the patient showed any signs of instability (no such events were reported). The second was a young thoracic surgeon (working experience less than 1 year) who performed the ultrasound examination (UR). The third was a highly experienced thoracic surgeon (working experience more than 20 years) who was in charge of locating the intercostal space through traditional manipulation methods (MR). The fourth was the final investigator (IR) who was in charge of verifying the intercostal space under direct vision through thoracoscopy.

After written consent was obtained, anesthesia was inducted for all patients. All subjects were monitored by electrocardiography, pulse oximetry, invasive blood pressure recordings, and bispectral index values. For fluid supplementation, 1-3 ml/kg/h crystalloid was administered. Midazolam 0.05 mg/kg, sufentanil 0.5 μg/kg, etomidate 0.2 mg/kg, and cisatracurium 0.2 mg/kg were intravenously administered for induction, followed by mechanical ventilation under double-lumen bronchial intubation; then, the patients were placed in a lateral position for surgery.

In this study, a single-port thoracoscopic lobectomy was performed. The incision port was determined by a fixed thoracic team, which was independent of this study. There was no specific requirement for how this team should determine the intercostal space required. The location of the incision was evaluated by the UR and MR. All operations were performed by the fixed thoracic surgeon team. After induction, all patients were placed in a lateral position. The location of the incision port was then evaluated by the UR, MR, and IR.

The positioning route was basically the same between the MR and the UR. The main difference between the two routes was the instrument adopted; one route was based on the hand, and the other was based on ultrasound. Briefly, the UR entered the operating room and determined which intercostal space he or she wanted to judge by ultrasound. The UR left the operating room when he or she determined a location. Then, the MR entered the operating room and determined which intercostal space he or she wanted to judge by hand. After completing his evaluation, the MR left the operating room, and the operation was started by the thoracic team. The IR entered the operating room and determined which intercostal space the incision port was actually in under direct vision with a thoracoscope. Conclusions made by the IR were recognized as the gold standard.

#### Positioning by the UR

**Fig. 1:**
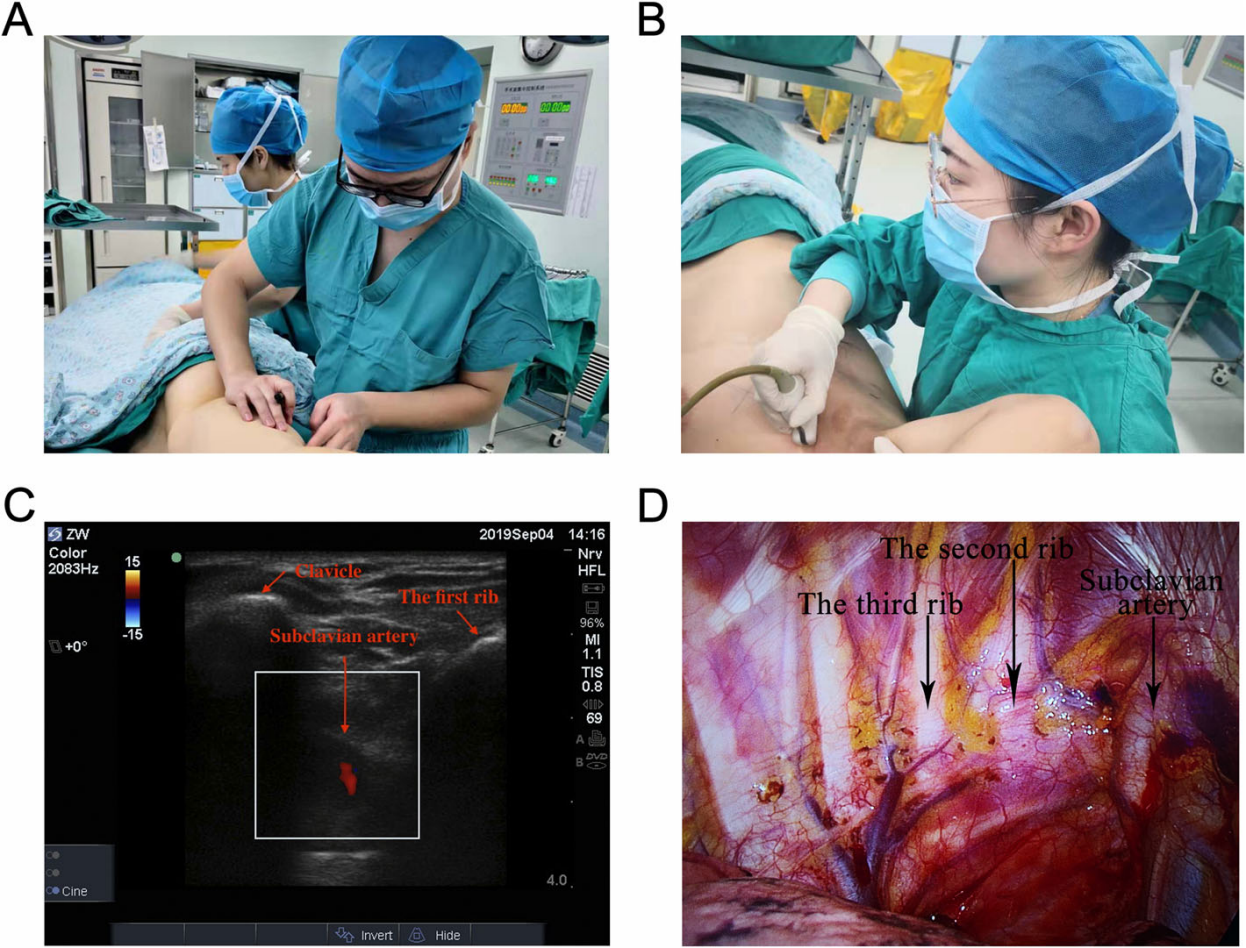
Intraoperative illustration showing the positioning of MR, UR, and IR. Intercostal space was located by manipulation (**a**) and ultrasound (**b**) respectively. As shown by the arrows, the clavicle, subclavian arteries, and the first rib could be easily found by ultrasound (**c**). The final investigator counted the ribs with the help of the thoracoscope (**d**)

Previous studies suggest that a minimum of 25 to 50 examinations are needed for point-of-care ultrasound training for other topics in both the emergency department and the intensive care unit. A training curriculum of point-of-care ultrasound for the young thoracic surgeon was provided before the study started, which required a minimum of 50 examinations to complete training [12]. After the incision approach was determined, the thoracic team left the operating room. The UR went into the operating room and started his evaluation. The UR performed his examination using the Philips IU-22 machine with a 12-MHz linear probe (shown in Fig. 1b). First, the probe was placed at the clavicle. Scanning was performed from the midline of the clavicle in a short-axis plane. The clavicle and subclavian arteries were first displayed on the screen. The color model could be useful for confirming the subclavian artery. The first rib, adjacent to the subclavian artery, could be easily found (shown in Fig. 1c). The recognition of the first rib was important. The intercostal space below the first rib is the first intercostal space. Then, the probe was positioned downward until the probe approached the location of the surgical incision. Finally, the UR made his judgment on which intercostal space the surgical incision would be made in. Then, the UR left the operating room. The time required by the UR was recorded.

#### Positioning by the MR

After the UR left the operating room, the MR went into the operating room and started his evaluation. The manipulation method, which is based on anatomic markers, was performed as follows (shown in Fig. 1a). The forward protrusion, where the manubrium and the mesosternum meet, was recognized as the sternal angle. The sternal angle, attached by the second rib, could be felt by hand. The intercostal space below the second rib is the second intercostal space. After the location of the second intercostal space was confirmed, the MR continued feeling the intercostal space downward until he or she approached the location of the surgical incision. Finally, the MR made his judgment on which intercostal space the surgical incision approach would be made in. Then, the MR left the operating room. The time required by the MR was recorded.

#### Positioning by the IR

After the UR and the MR completed their evaluations, the fixed thoracic team went into the operating room and started the operation. The standard method to confirm the location of the intercostal space was defined as counting and recognizing the ribs under direct vision after the thoracic cavity was opened. The final investigator went into the operating room and started his evaluation. After the thoracoscopic lens was placed into the thoracic cavity, the final investigator counted the ribs with the help of the thoracoscope (shown in Fig. 1d). After the first rib and the subclavian artery was confirmed, the final results of where the incision was made could be easily finally verified. The conclusion made by the final investigator was recognized as the gold standard.

#### Data acquisition

Gender, age, body mass index, and height, and time required by the UR and MR were recorded. The results provided by the IR were regarded as the gold standard. The results provided by the UR, and UR were scored as “Yes” or “No.”

#### Statistical analysis

According to the results of the preliminary experiment, the detection rates for the correct intercostal space with manipulation and ultrasound were 96.6% and 89.7%, respectively. The difference between the two methods was 6.9%, and the inconsistency rate was 10.3%. Assuming an *α* of 0.05 and a power value of 0.80, the McNemar method was used to test the differences in detecting the location of the intercostal space, and the minimum sample size was estimated to be 177 patients. Considering the data loss factor, a total of 200 patients were included in this study.

The diagnostic performance was evaluated with sensitivity, specificity, positive, and negative predictive value, and accuracy. All proportions were tested using a chi-square test. The confidence intervals (CIs) of the abovementioned parameters were calculated with the Pearson method. The inter-relationships between ultrasound and the gold standard and between manipulation and the gold standard were analyzed with Spearman correlation coefficients. The data were presented as means and standard deviations or medians and quartiles. The SPSS 22.0 software was used for the analysis, and the statistical data were analyzed with the chi-square test; *p* < 0.05 was considered statistically significant.

## Results

Data were collected from 200 subjects, and 20 subjects were excluded from the study (2 subjects had subcutaneous emphysema, and the operative routes were changed from a single port to other approaches in the other 18 subjects). Finally, a total of 180 subjects were included in the study. The patient demographic data are summarized in Table 1.

**Table 1:** Patient demographics

ASA class (total *n* = 180)	
I	38
II	125
III	17
Gender (male/female)	106/74
Age (year)	55 ± 13
Height (cm)	165.3 ± 7.2
Weight (kg)	66.4 ± 10.2
BMI	24.3 ± 3.2

Data are presented as total count or mean ± SD

The location of the incision was evaluated by two independent researchers (MR and UR). Final decisions were made through direct vision by IR, and these decisions were taken as the gold standard. The results of the two methods are shown in Table 2.

**Table 2:** Positioning results: manipulation vs. gold standard and ultrasound vs. gold standard

	Gold standard			
		+	−	Total
Manipulation	+	154	7	161
	−	16	3	19
Total		170	10	180
Ultrasound	+	148	4	152
	−	22	6	28
Total		170	10	180

There were no differences between manipulation and ultrasound-based positioning in terms of accuracy, which was 87.2% (95% CI 0.871-0.873) and 85.6% (95% CI 0.854-0.857), respectively (*p* > 0.05). Further details are shown in Table 3.

**Table 3:** The accuracy of manipulation vs. the accuracy of ultrasound % (95% CI)

Method	Accuracy
Ultrasound	0.856 (0.854-0.857)
Manipulation	0.872 (0.871-0.873)
χ^2^	0.196
*p*	0.66

Manipulation positioning showed a sensitivity of 90.6% (95% CI 0.862-0.950) and specificity of 30% (95% CI 0.016-0.584), while ultrasound positioning showed a sensitivity of 87.1% (95% CI 0.820-0.921) and specificity of 60% (95% CI 0.296-0.904). Compared with that of manipulation positioning, the specificity of ultrasound was higher (*p* < 0.05). No differences in PPV and NPV were found between manipulation and ultrasound positioning (*p* > 0.05). Further details are shown in Table 4.

**Table 4:** Test characteristics of manipulation vs. ultrasound % (95% CI)

Method	Sensitivity	Specificity	PPV	NPV
Ultrasound	0.871 (0.820-0.921)	0.600 (0.296-0.904)	0.974 (0.948-0.999)	0.214 (0.062-0.366)
Manipulation	0.906 (0.862-0.950)	0.300 (0.016-0.584)	0.957 (0.925-0.988)	0.158(−0.006-0.322)
*χ*^2^	1.11	32.73	0.78	1.86
*p*	0.29	0.001	0.38	0.17

*NPV* negative predictive value, *PPV* positive predictive value

According to the consistency test, the inter-relationships between ultrasound and the standard method and between manipulation and the standard method were consistent (*p* < 0.05). The details are shown in Table 5.

**Table 5:** Results of the consistency test

Method	*p*	Kappa value
Ultrasound vs. gold standard	0.000	0.255
Manipulation vs. gold standard	0.039	0.145

**Fig. 2:**
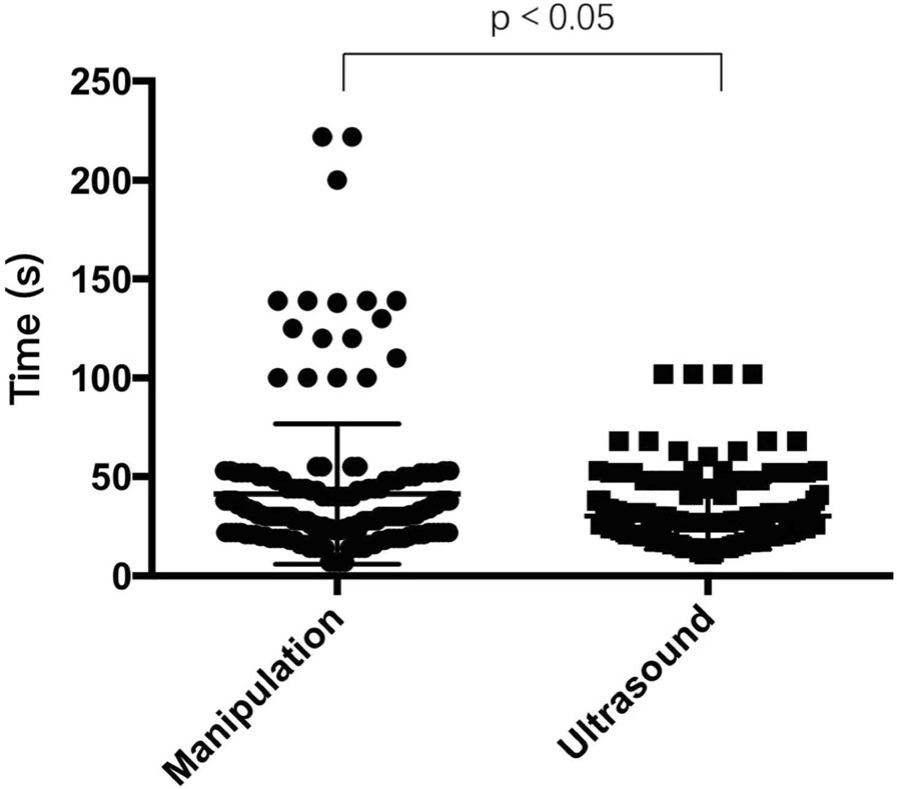
Comparison of the time required by the ultrasound and manipulation methods. The time required by UR and MR were 27 s (20 s, 33 s) and 30 s (22 s, 44 s), respectively. Since the data were not normally distributed, the data were analyzed by the Mann-Whitney *U* test; the time required by ultrasound was significantly shorter than that required by manipulation (*p* = 0.001 < 0.05)

The time required by UR and MR were 27 s (20 s, 33 s) and 30 s (22 s, 44 s), respectively. Since the data were not normally distributed, the data were analyzed by the Mann-Whitney *U* test; the time required by ultrasound was significantly shorter than that required by manipulation (*p = 0.001* < 0.05). The data are shown in Fig. 2.

## Discussion

To the best of our knowledge, this is the first study to evaluate the accuracy of ultrasound in locating an intercostal space for single-port thoracoscopic surgery. Our study showed that there was no difference between the accuracies of manipulation and ultrasound. The subsequent consistency test showed that both manipulation and ultrasound have good consistency with the gold standard. Currently, intercostal positioning relies more on manipulation than on ultrasound. Generally, the results from high-level surgeons with more experience were more reliable. Therefore, low-level surgeons with less experience have much more to learn. However, based on our results, after a relatively short learning period with ultrasound, even a young surgeon with minimal experience could obtain comparable accuracy. The use of ultrasound can equalize the level of less experienced doctors and flatten the learning curve.

One detail that needs our consideration is that the accuracy of ultrasound was not 100%. In this study, ultrasound positioning relied on the recognition of the first rib. Since the clavicle and the first rib are closely adjacent, there is a possibility that the second rib is mistaken as the first rib under ultrasound, resulting in positioning errors. However, the ultrasound-guided method still has advantages over the manipulation method, including being intuitive, objective, and visual.

Furthermore, no difference in sensitivity was found between manual localization and ultrasound localization, which was 0.906 and 0.871, respectively. This study demonstrated that the specificity of manipulation localization was significantly lower than that of ultrasound. Manipulation localization is susceptible to operator experience and thus is subjective. Meanwhile, manipulation localization relies heavily on anatomical markers, such as the sternal angle plane, which is typically connected to the second rib, or the subscapular angle inferior scapulae, which typically points to the seventh rib. However, anatomic variations, which are objective, may definitely affect the accuracy of manipulation positioning. For example, in some cases, the sternal hilt is very long, and the sternum angle is directly connected to the third rib. There are also other methods for positioning the 12th rib. However, the 12th rib is too short to confirm in some patients or is even absent; in rare cases, some people have a 13th rib, which would definitely cause errors [7]. Obviously, the probability of errors with manipulation positioning can increase due to anatomical variations. In addition, the difficulty of manipulation might be increased by other factors, such as obesity, the female sex, and fat thickness [8, 9]. In contrast, anatomical markers can be easily visualized by using ultrasound. Ultrasound is characterized as an objective method. In contrast, there is a large degree of subjectivity in manipulation localization, which may be the reason why the specificity of manipulation localization is lower than that of ultrasound localization in this study.

This study showed that the time required by ultrasound was shorter than that by required manipulation. The shortened evaluating time accelerated the working efficiency in the operating room. Moreover, the variability in the time required for ultrasound-guided positioning was small, while that of manual positioning was large, indicating that the manual localization method is more susceptible to interference by operator experience and patient factors, while the ultrasound localization method was more stable.

The study has the following shortcomings:

1. There are confounding factors in this study. In clinical practice, it is often more difficult to locate the intercostal space in obese patients, women, and patients with narrow intercostal spaces. All of these confounding factors were not excluded in this study. Although the sample size was determined by previous preliminary experiments, considering the universality of the results, some special populations failed to be excluded.
2. Only subjects who underwent single-port thoracoscopic surgery were enrolled. Whether our conclusion is suitable for double-port or three-port thoracoscopic surgery requires further study.
3. As discussed above, several manipulation approaches could be applied to determine the location of the intercostal space. Only one approach was studied, and the differences between ultrasound and manipulation for the same surgery were compared. There is uncertainty about the comparability of other manipulation approaches and ultrasound for the same surgery.

In summary, compared with the manipulation method, the ultrasound-guided method could be accurately applied to locate the intercostal space for single-port thoracoscopy surgery. Ultrasound requires less time than manipulation.


**Additional file 1: Video S1.**



## Data Availability

The datasets used and analyzed during the current study are available from the corresponding author on reasonable request.

## Abbreviations

ASA: American Society of Anesthesiologists
BMI: Body mass index
NPV: Negative predictive value
PPV: Positive predictive value
